# Cognitive frailty and its influencing factors in the elderly rural community residents of inner Mongolia, China

**DOI:** 10.1186/s12889-026-26230-w

**Published:** 2026-01-12

**Authors:** Lanjun Li, Xingbo Qu, Zhongshuang Ren, Yan Zhang, Lixia Hao, Yu Liang, Riletemuer Hu

**Affiliations:** 1 Rehabilitation Medicine Department, The Affiliated Hospital of Inner Mongolia Medical University, Hohhot, China; 2 Inner Mongolia Medical University, Hohhot, China

**Keywords:** Cognitive frailty, Aging, Elderly, Frailty, Community-dwelling

## Abstract

**Objectives:**

As a subtype of frailty, cognitive frailty (CF) has great significance on adverse outcomes in the elderly. The purpose of this study was to examine the prevalence of CF and the factors that contribute to it in an older population residing in rural communities in Inner Mongolia, China. Additionally, we aimed to identify potentially modifiable factors associated with CF to inform early intervention strategies for this population.

**Methods:**

This was a cross-sectional survey of older people living in rural communities in Inner Mongolia, China (*n* = 885), who had not been initially diagnosed with dementia(September to October 2024). According to the questionnaire results, the participants were divided into three groups: CF (cognitive frailty), PCF (pre-cognitive frailty), and Robust. Using the Mini-Mental State Examination (MMSE < 25) scale, elderly individuals were divided into two categories: non-cognitive impairment (NCI) and cognitive impairment (CI).The FRAIL Scale Phenotype (FP ≥ 3) scale was used to measure non-physical frailty (NPF) and physical frailty (PF). Thus, the participant groups were defined as follows: PCF (NPF + CI or PF + NCI), CF (PF + CI), and Robust (NPF + NCI).

**Results:**

The prevalence of CF was 9.27%. In the ordinal regression analysis, lower educational attainment was associated with higher risk, with Illiterate (OR = 5.378, 95% CI: 3.607–8.019, *p* < 0.001) and Primary education (OR = 1.689, 95% CI: 1.180–2.416, *P* = 0.004) categories showing significant effects. Participants with a family income of 10,000–19,999 yuan had higher odds (OR = 1.734, 95% CI: 1.193–2.520, *P* = 0.004). Without exercise was positively associated with higher risk (OR = 1.514, 95% CI: 1.084–2.114, *P* = 0.015), while greater grip strength was inversely associated (OR = 0.977, 95% CI: 0.957–0.998, *P* = 0.030). Additionally, higher scores on the simple five-item scoring questionnaire (SARC-F) significantly increased the odds (OR = 1.592, 95% CI: 1.432–1.771, *P* < 0.001).

**Conclusions:**

This study reports the prevalence of CF in a rural community of Inner Mongolia. The data showed the relationships between CF and sociodemographic factors and physical condition. This study highlights the importance of comprehensive geriatric health care for elderly residents of Inner Mongolia.

**Trial registration:**

KY2024060.

**Supplementary Information:**

The online version contains supplementary material available at 10.1186/s12889-026-26230-w.

## Introduction

Aging is the gradual and total physiological depletion of an organism’s reserves, which reduces its capacity to produce adaptive responses and maintain homeostasis [1]. The incidence of cognitive impairment and frailty are higher in the elderly population, resulting in adverse health outcomes, and contributing to the huge pressure on social medical security.

Cognitive impairment (CI) refers to a measurable decline in one or more cognitive domains—such as memory, attention, language, or executive function—that interferes with daily activities, regardless of whether structural brain changes are present [2, 3]. CI encompasses conditions ranging from mild cognitive impairment to dementia. This can have an impact on an individual’s day-to-day functioning or social interactions [2]. Within the category of CI is dementia and mild cognitive impairment (MCI). Furthermore, vascular illness, metabolic disorders, trauma, infectious infections, depression, and polypharmacy have all been linked to the development of cognitive decline [3].

Frailty is a complex clinical syndrome, and its pathogenesis remains unclear. It is not only closely related to aging, but also to poor outcomes in the elderly. The elderly can be divided into the following frailty phenotypes: frail, pre-frail, and robust. The state of frailty is reversible; therefore, early diagnosis and early treatment are very important in elderly people. As research continues to deepen, more modern definitions of frailty have considered at least three domains, including physical, cognitive, and psychosocial characteristics, and maintaining the intricate links between them. Thus, frailty is no longer solely thought to be tied to physical status [4].

According to the International Consensus Panel [5, 6], cognitive frailty (CF) is defined as the co-existence of physical frailty and cognitive impairment in the absence of dementia or other underlying brain diseases. People with CF are at a higher risk of experiencing negative health outcomes in their later years, such as dementia, falls, hospitalization, and mortality, in comparison to those who only have physical frailty or CI [7]. Recent epidemiological investigations and meta-analyses have reported a broad range in the prevalence of CF among older adults in China. A national systematic review by Qiu et al. [8] estimated an overall prevalence of 9%, whereas a more recent pooled analysis by Xie et al. [9] reported a higher prevalence of 18.9%, particularly in rural populations. Furthermore, data from Wang et al. [10] revealed that CF occurs approximately 1.6 times more frequently in rural than in urban regions, reflecting disparities in healthcare access, socioeconomic conditions, and physical-activity opportunities. This puts a huge burden on individuals, families and even society. The concept of cognitive decline was first put forward by Kelaiditi et al. [6] in older adults, taking into account the co-existence, in the absence of dementia or underlying brain illnesses, of MCI (Clinical Dementia Rating, CDR = 0.5) and physical frailty. This idea demonstrated that frailty appeared to precede and predict the development of dementia, and that subclinical cognitive pathology may have begun as a component of frailty years before overt dementia arose [11]. Regarding reversibility, Ruan et al. [1] identified further differences between possibly reversible and reversible cognitive fragility. Subjective cognitive decline (SCD) and positive neurodegenerative biomarkers point to the former, while motor cognitive impairment points to the latter.

The rural elderly population in Inner Mongolia faces unique risk factors, including limited healthcare access, long winters restricting physical activity, and a traditional high-fat diet. These factors may contribute to higher frailty and cognitive impairment risks compared to urban populations, underscoring the importance of studying this underrepresented region [12]. There is no clear conclusion regarding the influencing factors of CF, and no relevant research has been conducted in Inner Mongolia. To offer helpful suggestions, this study primarily focuses on the epidemiology and contributing causes of CF in an older population residing in rural communities in Inner Mongolia, China.

## Materials and methods

The study participants included elderly persons who had lived for more than 10 years in a community in the Inner Mongolia Autonomous Region, China. Each individual or legally recognized representative signed an informed consent form. Every step of our study is according to Declaration of Helsinki and International Ethical Guidelines for Human Biomedical Research.

### Choice of address

This study selected a community in the Wulagai Management District of Xilin Gol League, Inner Mongolia Autonomous Region, as the research community. Northeast of the Gol League of Xilin, it is located at the junction of Xi-League, Xingan League, and Tongliao. The community health center provides comprehensive primary healthcare services, including chronic disease management, blood tests, and electrocardiography, which ensures reliable clinical data collection. The crowd cooperation and follow-up rate of the population was high, generally reaching > 90%. The community workers have the corresponding qualifications. For elderly residents in the community, a database was established to systematic record and manage physical examination results and medical records. In this study, we conducted a cross-sectional secondary analysis of this established community medical database, and all variables were extracted from the database.

### Study population

Our study included all members of the community over the age of 60 years. The participants met the following inclusion criteria: (i) consenting older adults of both sexes; (ii) having spent > 10 years residing in the specified community; (iii) being conscious and able to communicate with normal language, and (iv) could complete the relevant tests independently. The following participant exclusion criteria were applied: (i) given a dementia diagnosis, such as Alzheimer’s disease, and (ii) had a serious physical disease or in the acute phase of the disease.

### Features of the socio-demographic group

Sociodemographic data were collected on gender, age, height, weight, grip strength, ethnicity, educational level, marital status, family income, marital status, smoking, drinking, excising, body mass index (BMI), and so on.

Diagnosis of hypertension was established as follows: systolic blood pressure ≥ 140 mmHg, diastolic blood pressure ≥ 90 mmHg (Blood pressure measured twice or more on different days exceeds the above range. No exercise, smoking, or drinking alcohol or coffee in the 30 min before the measurement), or the diagnosis of hypertension confirmed in medical records or current pre- scription of antihypertensive medications.

Exercising: Patients were considered exercising if they reported walking or performing physical activities for ≥ 150 min, divided into two or three periods (each one with at least 45 min) weekly.

### Functional evaluation

We used the activities of daily living scale (ADL) and the simple five-item scoring questionnaire (SARC-F) scale for the functional evaluation of the elderly participants.

ADL scale: There were 14 questions in this scale, and each question is scored based on the subject’s response: 1 point indicates people can do it themselves; 2 points indicate some difficulty; and 3 points mean people need help. A total score of 14 is considered normal, and a score greater than or equal to 15 indicates different levels of physical dysfunction.

SARC-F Scale: This scale was composed of five parts: strength, walking aid, standing up from a chair, climbing stairs, and falling. On a scale of 0–10, physical activity levels decline as the scores rise [13]. At present, it is generally accepted that a score of ≥ 4 points can be diagnosed as sarcopenia [14].

### Evaluation of cognitive abilities

Utilizing the Mini-Mental State Examination (MMSE) scale, cognitive function was evaluated [15]; this scale was translated into the Chinese language [16]. The MMSE is a 30-point questionnaire, encompassing five distinct dimensions: language, orientation, focus, memory, and visual-spatial abilities. The severity of the CI increases with a lower score. Within this study, CI was defined as a score < 25, and non-cognitive impairment (NCI) as a score > 25 [17, 18].

### Assessment of physical frailty

The International Nutrition, Health, and Work Group’s experts proposed the FRAIL Scale [19] in 2008. The frailty index and phenotype serve as the foundation for this scale.

The FRAIL Scale is comprised of 36 items. A portion of them were chosen from the Medical Outcome Study Short Form, and some items were selected from disease and weight loss. Every item on the resulting five-item scale is worth one point. This scale has a score range of 0 to 5. Frailty would be identified with a score of 3 or above; a score of 1–2 was diagnosed as pre-frailty, and a score of 0 was diagnosed as robust.

### CF

The concept of CF provided by Kelaiditi et al. (2013) was used for the assessment of CF [6]. In line with this framework, we conceptualized cognitive frailty as the coexistence of physical frailty and cognitive impairment in the absence of dementia; therefore, individuals with a clinical diagnosis of dementia were not eligible for CF classification in our study. According to the evaluation of cognitive abilities and assessment of physical frailty, the population was divided into three categories: CF, pre-CF (PCF), and Robust. The detailed grouping criteria are shown in Table 1.

**Table 1:** Operationalization of cognitive frailty characteristics

	Cognitive frailty	Pre-cognitive frailty		Robust
Physical Frailty	PF	NCI	PF	NPF
Cognitive Impairment	CI	CI	NPF	NCI

*NPF* Non-Physical Frailty, *PF *Physical Frailty, *NCI *No-Cognitive Impairment, *CI *Cognitive Impairment

### Statistical analysis

All data were double-checked and recorded by two people (after formal training and obtaining a certificate) and the SPSS (Statistical Package for Social Sciences) 25.0 software was used for statistical analysis. The mean ± standard error (x ± s) was used to describe the distribution of the measurement data that followed the normal distribution, and the median (Q1, Q3) was used for non-normally distributed data. The ANOVA (F) and rank sum test (Z) were used for comparisons across different groups. Frequencies and percentages were employed to characterize the counting data, and the χ2 test was utilized to compare the groups. In the univariate analyses, these variables with *p* < 0.05 were considered significant variables.

Given the ordered categorical nature of cognitive-frailty status (Robust*→*Pre-CF*→*CF), we applied an ordinal logistic regression model using the proportional-odds approach. The Brant test was used to verify the proportional-odds assumption. To assess collinearity, the Variance Inflation Factor (VIF) was calculated for all predictors. Model adequacy and explanatory power were evaluated using the Akaike Information Criterion (AIC), Bayesian Information Criterion (BIC), and Nagelkerke R^2^. Additionally, age-stratified ordinal logistic models were performed for participants aged 60–70 and > 70 years to examine the stability of associations across age groups. The variables that comprised the final multinomial logistic model were those that showed statistical significance (*p* < 0.05) in each model.

## Results

Of 885 elderly study participants, 82 (9.27%) had CF, 372 (42.03%) had PCF, and 431(48.70%) were Robust. The participants were aged between 60 and 88 years old (mean: 67 years). The mean age of the CF group was 70.5 years, which was significantly higher than that for the PCF group (68 years) and the Robust group (67 years) (*p* < 0.001). Furthermore, 59 females (12.11%) and 23 males (5.78%) had CF. Of the CF participants, 52 were illiterate (18.71%) and 15 had a family income of < 10,000 yuan (12.00%).

In elderly participants, hypertension (59.43%, *n* = 526) and coronary heart disease (23.05%, *n* = 204) were significantly associated with CF (*p* < 0.001). In addition, in CF participants, traumatism (21.62%, *n* = 8) was an important characteristic among CF participants. Finally, elderly participants’ marital status and level of functioning were not substantially (*p* > 0.05) linked to CF. Further detail is shown in Table 2.

**Table 2:** Basic characteristics with cognitive frailty (CF) categories

Characteristic		CF (*n* = 82)	Pre-CF (*n* = 372)	Robust (*n* = 431)	Total (*n* = 885)	test values	*P*-value
Age		70.5 (66, 75)	68 (64, 72.75)	67 (63, 71)	67 (64.72)	Z = 43.553	<0.001
Sex	Male	23 (5.78)	150 (37.69)	225 (56.53)	398	χ2 = 21.849	<0.001
	Female	59 (12.11)	222 (45.59)	206 (42.30)	487		
Education	Illiterate	52 (18.71)	168 (60.43)	58 (20.86)	278	χ2 = 152.751	<0.001
	Primary	20 (6.43)	125 (40.19)	166 (53.38)	311		
	Higher education	10 (3.38)	79 (26.69)	207 (69.93)	296		
Marital status	Spinsterhood	2 (16.67)	5 (41.67)	5 (41.67)	12	χ2 = 6.728	0.347
	Married	62 (8.62)	294 (40.89)	363 (50.49)	719		
	Widowed	17 (12.32)	66 (47.83)	55 (39.86)	138		
	Divorce	1 (6.25)	7 (43.75)	8 (50.00)	16		
Family income (yuan)	<10,000	15 (12.00)	55 (44.00)	55 (44.00)	125	χ2 = 24.264	<0.001
	10,000–19,999	26 (12.50)	104 (50.00)	78 (37.50)	208		
	20,000–34,999	15 (7.77)	86 (44.56)	92 (47.67)	193		
	≥ 35,000	26 (7.24)	127 (35.38)	206 (57.38)	359		
Hypertension	No	20 (5.57)	155 (43.18)	184 (51.25)	359	χ2 = 9.894	<0.001
	Yes	62 (11.79)	217 (41.25)	247 (46.96)	526		
Coronary heart disease	No	56 (8.22)	282 (41.41)	343 (50.37)	681	χ2 = 5.423	<0.001
	Yes	26 (12.75)	90 (44.12)	88 (43.14)	204		
Operation	Yes	30 (9.40)	127 (39.81)	162 (50.78)	319	χ2 = 1.041	0.591
	No	52 (9.19)	245 (43.29)	269 (47.53)	566		
Traumatism	Yes	8 (21.62)	13 (35.14)	16 (43.24)	37	χ2 = 7.036	<0.05
	No	74 (8.73)	359 (42.33)	415 (48.94)	848		

Traumatism referred to a history of injury/trauma recorded in the community medical database (medical records and/or questionnaire), requiring medical attention (e.g., clinic visit or hospitalization). Operation referred to a history of surgery recorded in the community medical database (medical records), typically procedures requiring anesthesia and/or hospitalization

Regarding the physical state with CF categories (Table 3), grip strength showed significant differences between groups (*p* < 0.001). In addition, exercise had a significant impact on CF (*p* < 0.001). Different situations of drinking showed an effect across three groups in this study. Furthermore, the cognitively frail participants had a high ADL score (14, 14–26.25) and a high SARC-F score [2–6] compared to the other two groups.

**Table 3:** Physical state with cognitive frailty (CF) categories

Characteristic		CF (*n* = 82)	Pre-CF (*n* = 372)	Robust (*n* = 431)	Total (*n* = 885)	test values	*P*-value
BMI (kg/m^2^)		25.10 ± 3.75	24.71 ± 4.10	25.02 ± 5.05	24.9 ± 4.56	F = 0.534	0.586
Grip strength (average**)**		15.3 (12.18, 20.36)	19.75 (15.55, 24.53)	23.25 (18.65, 30.5)	20.75 (16.12, 27.45)	Z = 88.305	<0.001
Exercising	No	70 (10.92)	287 (44.77)	284 (44.31)	641	χ2 = = 20.245	<0.001
	Yes	12 (4.92)	85 (34.84)	147 (60.25)	244		
Smoking	Never	66 (10.20)	277 (42.81)	304 (46.99)	647	χ2 = 6.280	0.179
	Quitting	7 (7.45)	32 (34.04)	55 (58.51)	94		
	Yes	9 (6.25)	63 (43.75)	72 (50.00)	144		
Drinking	Never	75 (11.08)	301 (44.46)	301 (44.46)	677	Χ2 = 29.712	<0.001
	Sometime	2 (1.75)	36 (31.58)	76 (66.67)	114		
	Often	3 (14.29)	6 (28.57)	12 (57.14)	21		
	Every day	2 (2.74)	29 (39.73)	42 (57.53)	73		
ADL score		14 (14, 26.25)	14 (14, 14)	14 (14, 14)	14 (14, 14)	Z = 85.072	<0.001
SARC-F score		4 (2, 6)	0 (0, 2)	0 (0, 0)	0 (0, 1)	Z = 186.772	<0.001

The number and prevalence of patients in different ages and genders was showed in Table 4. We can conclude that the higher age, the greater the prevalence, and in different age groups, the prevalence of female is higher than that of male.

**Table 4:** The number and prevalence of patients in different ages and genders

Age	Male	Female	Total
60–69	12(2.20%)	23(4.22%)	35(6.42%)
70–79	8(2.69%)	30(10.10%)	38(12.79%)
≥ 80	3(6.97%)	6(13.95%)	9(20.92%)
Total	23(5.78%)	59(12.11%)	82(9.27%)

**Fig. 1:**
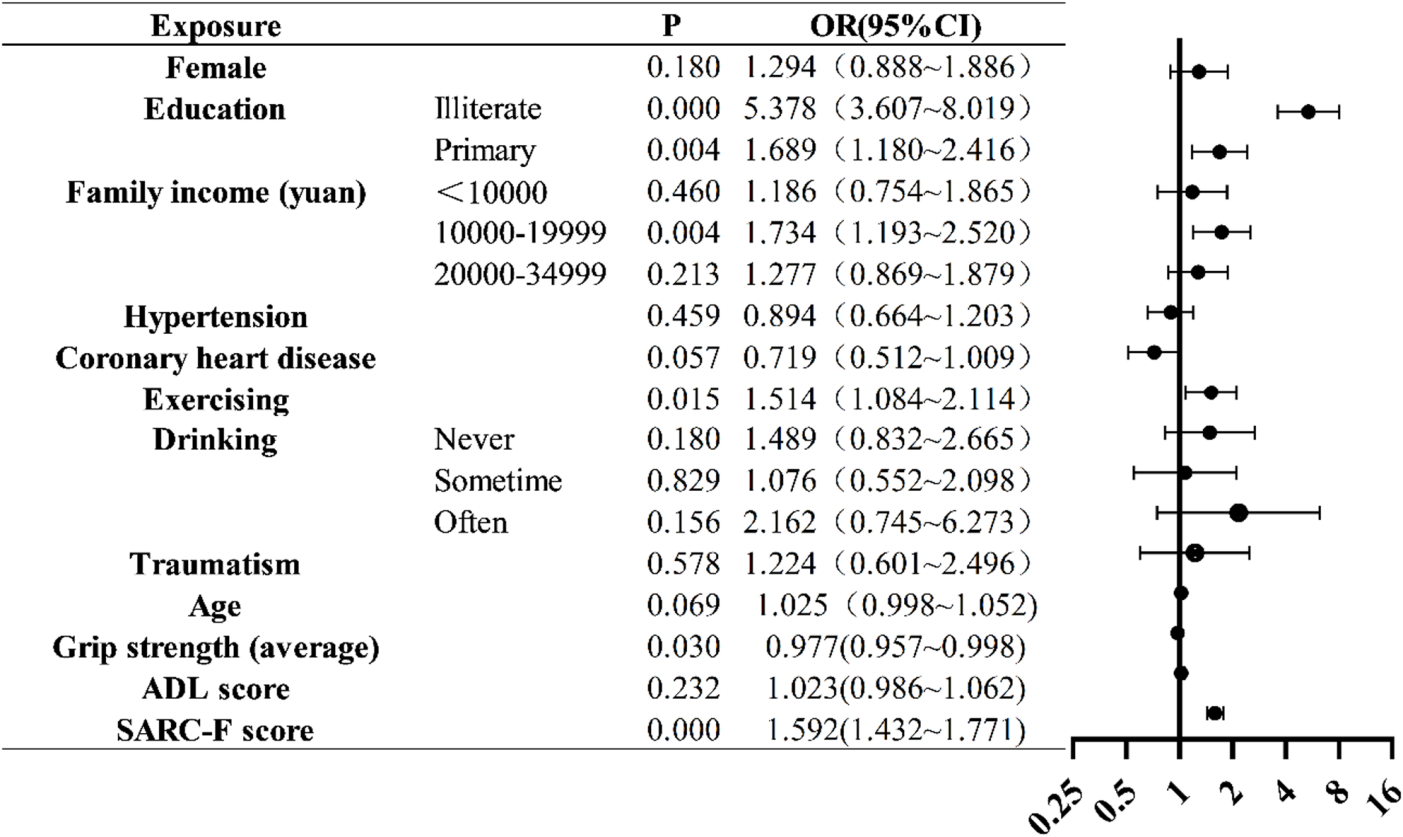
Ordinal logistic regression of CF. The reference for education was higher education; The reference for family income was ≥ 35,000; The reference for hypertension, coronary heart disease, and exercising was “Yes”; The reference for drinking was everyday; The reference for traumatism was “No”

Model diagnostics confirmed the suitability and stability of the ordinal logistic regression. The Brant test (*p* = 0.332) indicated that the proportional-odds assumption was not violated. No multicollinearity was detected (all VIF < 2). The model demonstrated good adequacy (AIC = 1319.159; BIC = 1357.63) and moderate explanatory power (Nagelkerke R^2^ = 0.41) (Supplementary TableS1). Lower educational attainment was associated with higher risk, with Illiterate (OR = 5.378, 95% CI: 3.607–8.019, *p* < 0.001) and Primary education (OR = 1.689, 95% CI: 1.180–2.416, *P* = 0.004) categories showing significant effects. Participants with a family income of 10,000–19,999 yuan had higher odds (OR = 1.734, 95% CI: 1.193–2.520, *P* = 0.004). Without exercise was positively associated with the higher risk (OR = 1.514, 95% CI: 1.084–2.114, *P* = 0.015), while greater grip strength was inversely associated (OR = 0.977, 95% CI: 0.957–0.998, *P* = 0.030). Additionally, higher SARC-F scores significantly increased the odds (OR = 1.592, 95% CI: 1.432–1.771, *P* < 0.001). Other factors, including sex, hypertension, coronary heart disease, drinking habits, traumatism, age, and ADL score, were not significantly associated (all *p* > 0.05) (Fig. 1 and Supplementary Table S2).

### Subgroup analysis

**Fig. 2:**
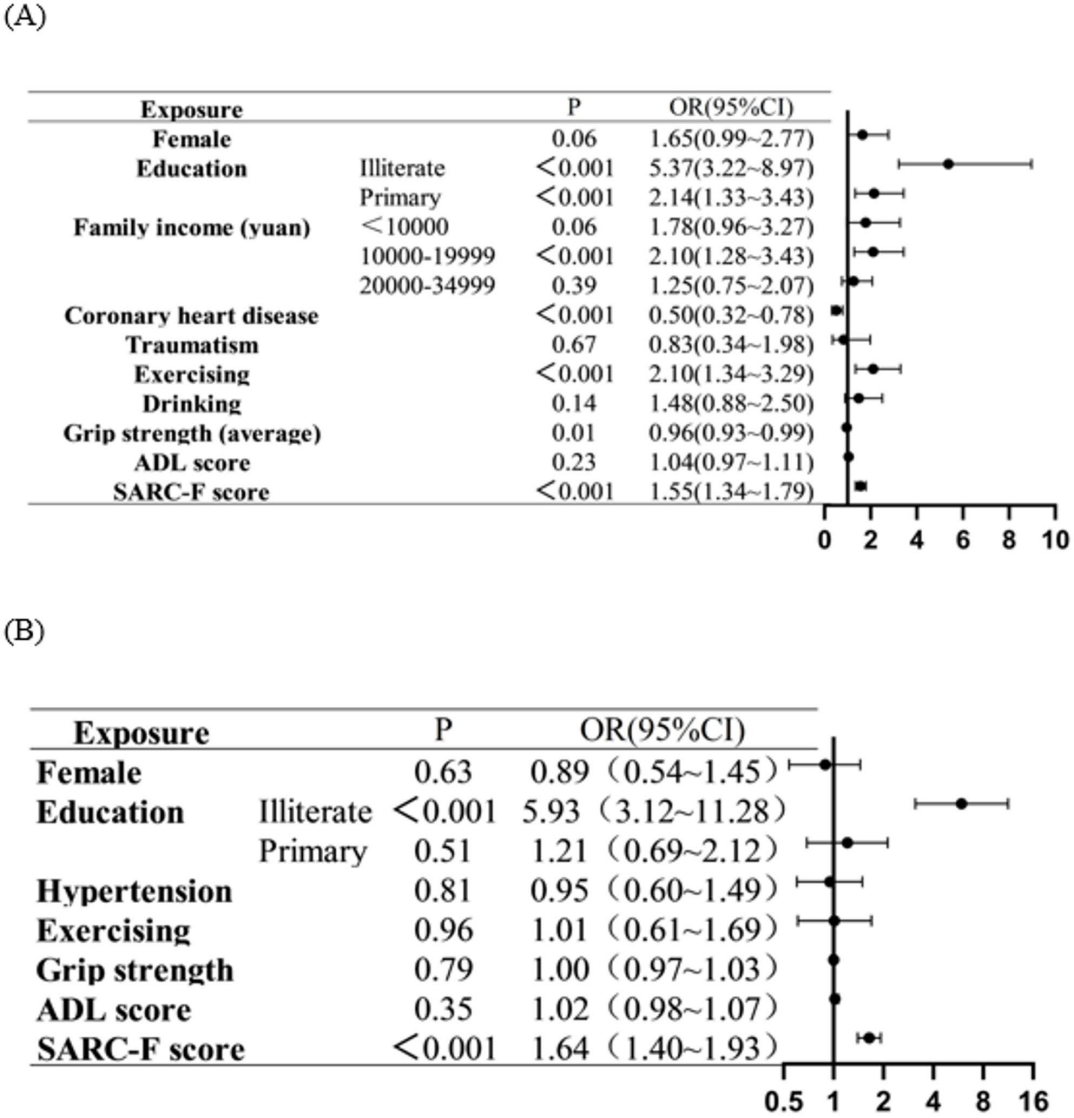
Ordinal logistic regression of CF by age stratification. **A** Aged 60-70;**B** Aged over 70. The reference for education was higher education; The reference for family income was ≥35000; The reference for hypertension, coronary heart disease, and exercising was “Yes”; The reference for drinking was everyday; The reference for traumatism was “Yes”

The subgroup analysis was conducted among participants aged 60–70 years old and participants who aged over 70 years old. The characteristics were shown in Supplementary Tables S3 and S4. The Brant test for both subgroups indicated proportional-odds assumption was not violated. Age-stratified ordinal logistic regression analyses were conducted to control for potential confounding by education and other sociodemographic variables (Supplementary Tables S5 and S6). In both age groups (60–70 years and > 70 years), lower education remained a strong independent predictor of cognitive frailty (illiterate: OR = 5.37 and 5.93, both *p* < 0.001) (Fig. 2). Model diagnostics confirmed no multicollinearity (all VIF < 2.0) (Supplementary Tables S7 and 8). These results demonstrate that the associations between age, physical function, and cognitive frailty persisted after adjustment for education.

## Discussion

In recent years, CF has attracted increasing attention regarding the significance of research on preventive therapies in aging [20]. Numerous studies have reported a 1% to 5% incidence of CF in community settings [21–23]; however, some studies have shown that the prevalence of CF ranges from 12.7% to 31.8% [5, 24–26]. Simultaneously, the pooled prevalence of CF was 9% (95% CI: 8–11%, I^2^ = 99.3%), according to a meta-analysis [8]. In the present study, we report that 9.27% of an elderly community in Inner Mongolia had CF. The number of elderly people in different studies and the difference in the measuring scales may account for the different prevalence rates reported. Notably, although CF has a conceptual definition, there is currently no universally agreed approach to operationalizing cognitive impairment and physical frailty in CF; prior studies have used different cognitive screening tools and cutoffs, as well as different frailty instruments, which introduces heterogeneity and limits direct comparability across studies. The primary conclusions of the present study were that a lower educational status, the presence of hypertension or sarcopenia, and no exercise could increase the risk of CF in elderly populations living in rural communities. These results are of great significance for the prevention and early intervention of CF in the elderly community in Inner Mongolia.

Education level has always been an important factor in improving the quality of life of the population. Education is a crucial tool for improving public health and well-being as it raises awareness and lessens the need for medical care [27]. The results of this study suggest that increasing educational attainment is a protective factor for CF, suggesting the importance of education in preventing adverse health outcomes in older age. The protective effect of education observed in our study supports the Cognitive Reserve Theory [28], which posits that higher educational attainment enhances neural efficiency and flexibility, enabling compensation for age-related or pathological brain changes. By fostering lifelong cognitive and social engagement, education contributes to both structural and functional brain reserve, helping preserve cognitive function despite neurodegeneration. In the context of cognitive frailty, higher education may mitigate the combined effects of physical frailty and cognitive impairment through improved health literacy, self-care, and proactive health behaviors. These findings highlight the importance of promoting lifelong learning and community-based cognitive engagement to reduce cognitive frailty risk in older adults.

Hypertension, as a common disease in the elderly population, is closely related to adverse outcomes in the elderly. Hypertension treatment and follow-up are easy to perform in the community. According to several studies, high blood pressure is frequently linked to cognitive decline and physical frailty [29]. Some studies have assessed the factors that affect CF in people with hypertension [30, 31]; however, our study showed that the progression of CF is closely related to hypertension. Persistent hypertension can induce cerebral small vessel disease (CSVD), resulting in microinfarcts, white matter hyperintensities, and reduced cerebral perfusion, which collectively accelerate cognitive decline and motor impairment. CSVD leads to white matter lesions, including leukoaraiosis and periventricular hyperintensities, that disrupt long-range neural connectivity essential for cognitive processing and gait control. In addition, cerebral microinfarcts, often clinically silent, accumulate over time and cause localized neuronal loss and cortical thinning, further compromising executive and memory functions. These microvascular alterations also weaken the the blood-brain barrier (BBB), increasing permeability to circulating inflammatory mediators and oxidative stress [32]. Recent experimental and clinical evidence suggests that IL-17-mediated neuroinflammation plays a central role in this process: elevated IL-17 promotes endothelial dysfunction, impairs BBB integrity, and activates microglia, leading to synaptic loss and neurodegeneration [32, 33]. These vascular and inflammatory pathways may underlie the bidirectional relationship between hypertension and frailty, providing a plausible biological explanation for their synergistic contribution to cognitive frailty in older adults. It is suggested that hypertension should be prevented and controlled in the elderly to prevent and delay CF.

As people age, their skeletal muscle mass decreases along with their muscle strength and/or physical activity level. This condition is known as sarcopenia, which is defined by a loss of muscle mass and function [34]. The SARC-F Scale is associated with sarcopenia; a higher score indicates more severe sarcopenia. At present, it is generally accepted that a score of ≥ 4 points can be diagnosed as sarcopenia. Prior research has demonstrated that sarcopenia is linked to a higher incidence of frailty and disability [8]. A meta-analysis found that sarcopenia increased the risk of MCI [35]. This study shows that sarcopenia is a risk factor for CF. Interventions for sarcopenia can also delay the development of CF. Research on the connection between sarcopenia and cognitive frailty in the Chinese community is still in the exploratory stage, and the specific mechanism still needs to be studied.

There may be benefits to exercise for sarcopenia and frailty [36]. Exercise therapies are crucial for managing CF without the use of pharmaceuticals [37]. According to a previous study, older persons with CF can benefit from high-speed resistance exercise training methods that enhance both their physical and cognitive abilities [37]. On the other hand, not much is known about the association between CF and exercise patterns (frequency, intensity, and timing). Our study only points to exercise as a protective factor for CF. In the future, the type and time of exercising should be planned in detail to provide more suitable exercise programs for elderly patients with CF.

This study had several limitations. First, because this study was based on secondary database analysis, validation using other frailty measures (e.g., FI-35) was not feasible. The FRAIL scale, although widely used, has shown moderate consistency (Kappa = 0.54–0.58) in Chinese older adult populations when compared with other frailty tools [38]. Meanwhile, the use of MMSE < 25 as a binary cutoff may yield false positives among participants with limited schooling. In future work, adopting education-adjusted cutoffs (e.g. ≤ 21–22) as suggested in Jia et al. [39] could reduce such misclassification. Second, we excluded participants with clinically diagnosed or severe dementia to align with the commonly accepted definition of cognitive frailty (i.e., the coexistence of physical frailty and cognitive impairment in the absence of dementia). Therefore, this exclusion should not be interpreted as underestimating the prevalence of cognitive frailty under the strict definition. However, excluding dementia cases may still introduce selection (range-restriction) bias by removing individuals at the extreme end of cognitive and physical impairment, which may limit the generalizability of our findings and prevent us from characterizing more severe phenotypes (e.g., dementia with frailty). This restricted range may also influence our estimates of association strength. Third, the exercise definition in this study (≥ 150 min per week, 2–3 sessions) did not fully comply with WHO [40] recommendations of bouts ≥ 10 min, which may have led to underestimation of physical activity levels. Because of the rigid requirement of ≥ 150 min per week in our dataset, short bouts of activity (< 10 min) were not captured, potentially underestimating total physical activity compared to WHO’s guidance that any duration counts toward the weekly total.

In addition to conventional risk factors, the regional context of Inner Mongolia may contribute to the distinct cognitive-frailty pattern observed. The region’s long, cold winters limit outdoor activity and social participation, while the traditional high-fat, high-salt diet increases metabolic and vascular risk. Limited access to specialized geriatric care and rehabilitation services in rural areas further exacerbates physical inactivity and delayed management of chronic conditions. Notably, although our study community had relatively well-functioning primary healthcare services, it may represent a better-resourced rural setting than some other areas in Inner Mongolia; therefore, the generalizability of our findings to the entire region should be interpreted with caution, particularly for communities with poorer access to care. These environmental, lifestyle, and healthcare factors may synergistically heighten susceptibility to both frailty and cognitive impairment. Future prevention strategies should therefore integrate culturally tailored interventions—such as seasonal activity programs, dietary counseling, and community-based cognitive engagement initiatives—adapted to the climatic and socioeconomic characteristics of Inner Mongolia.

## Conclusion

This is the first investigation into the variables that contribute to CF in the elderly population of Inner Mongolia. This study suggests that to reduce the prevalence of CF, it is necessary to improve the educational level of the population, encourage the elderly to increase the amount of exercise and prevent hypertension. This study shows that there is a close relationship between cognitive decline and muscle decay, which is worthy of further study in the future.

## Supplementary Information

Supplementary Material 1.

## Data Availability

We can share our research data on BMC public health (research data helpdesk), or you can sendE-mail:1977149269@qq.com.

## Abbreviations

CF: Cognitive frailty
PCF: Pre-cognitive frailty
MMSE: Mini-mental state examination
NCI: Non-cognitive impairment
CI: Cognitive impairment
FP: FRAIL Scale Phenotype
NPF: Non-physical frailty
PF: Physical frailty
OR: Odds ratio
CI: Confidence interval
SARC-F: Simple five-item scoring questionnaire
MCI: Mild cognitive impairment
CDR: Clinical dementia rating
SCD: Subjective cognitive decline
ADL: Activity of daily living scale
BMI: Body mass index
CIs: Confidence intervals
AIC: Akaike Information Criterion
BIC: Bayesian Information Criterion
VIF: Variance Inflation Factor
