# Supplementary material for: Cognitive frailty and its influencing factors in the elderly rural community residents of inner Mongolia, China
Source: BMC Public Health. 2026 Jan 12;26:522. doi: 10.1186/s12889-026-26230-w (PMC12888658; doi:10.1186/s12889-026-26230-w)
Supplement: Supplementary file 1 — Supplementary Material 1. [file 12889_2026_26230_MOESM1_ESM.docx]

Supplementary Table S1. The multicolinearity assessment among overall population

| **multicolinearity assessment** | | |
| --- | --- | --- |
| **model** | Tolerance | VIF |
| **age** | 0.81 | 1.23 |
| **sex** | 0.53 | 1.87 |
| **Education** |  | 1.26 |
| **Marital status** | 0.86 | 1.16 |
| **Family income (yuan)** | 0.84 | 1.19 |
| **Hypertension** | 0.90 | 1.11 |
| **Coronary heart disease** | 0.92 | 1.09 |
| **Grip strength (average)** | 0.62 | 1.61 |
| **Exercising** | 0.95 | 1.06 |
| **Drinking** | 0.75 | 1.34 |
| **ADL score** | 0.61 | 1.63 |
| **SARC-F score** | 0.59 | 1.70 |
| **Operation** | 0.94 |  |
| **BMI (kg/m2)** | 0.90 | 1.11 |
| **Traumatism** | 0.95 | 1.05 |
| **Smoking** | 0.79 | 1.27 |

**Supplementary Table S2.** The ordinal logistic regression among overall population

| **Exposure** | P | OR | 95%CI | |
| --- | --- | --- | --- | --- |
| **Robust** | 0.002 | 27.782 | 3.474 | 222.142 |
| **Pre-CF** | 0.000 | 778.617 | 92.493 | 6554.487 |
| **Female (vs. male)** | 0.180 | 1.294 | 0.888 | 1.886 |
| **Education (vs. Higher education)** |  |  |  |  |
| Illiterate | <0.001 | 5.378 | 3.607 | 8.019 |
| Primary | 0.004 | 1.689 | 1.180 | 2.416 |
| **Family income (yuan) (vs. ≥35000)** |  |  |  |  |
| **＜**10000 | 0.460 | 1.186 | 0.754 | 1.865 |
| 10000-19999 | 0.004 | 1.734 | 1.193 | 2.520 |
| 20000-34999 | 0.213 | 1.277 | 0.869 | 1.879 |
| **Hypertension (No vs. Yes)** | 0.459 | 0.894 | 0.664 | 1.203 |
| **Coronary heart disease (No vs. Yes)** | 0.057 | 0.719 | 0.512 | 1.009 |
| **Exercising (No vs. Yes)** | 0.015 | 1.514 | 1.084 | 2.114 |
| **Drinking (vs. Every day)** |  |  |  |  |
| Never | 0.180 | 1.489 | 0.832 | 2.665 |
| Sometime | 0.829 | 1.076 | 0.552 | 2.098 |
| Often | 0.156 | 2.162 | 0.745 | 6.273 |
| **Traumatism (Yes vs. No)** | 0.578 | 1.224 | 0.601 | 2.496 |
| **Age** | 0.069 | 1.025 | 0.998 | 1.052 |
| **Grip strength (average)** | 0.030 | 0.977 | 0.957 | 0.998 |
| **ADL score** | 0.232 | 1.023 | 0.986 | 1.062 |
| **SARC-F score** | ＜0.001 | 1.592 | 1.432 | 1.771 |

**Supplementary Table S3.** The characteristics among participants aged 60-70 years old

| **Characteristic** |  | **Robust (n=289)** | **Pre-CF (n=221)** | **CF (n=35)** | **Total** | **test values** | **P-value** |
| --- | --- | --- | --- | --- | --- | --- | --- |
|  |  |  |  |  | **(n=545)** |  |  |
| **Sex** | Male | 145（62.50） | 75（32.33） | 12（5.17） | 232 | χ2=14.553 | 0.001 |
|  | Female | 144（46.01） | 146（46.65） | 23（7.35） | 313 |  |  |
| BMI (kg/m**^2^**) |  | 24.80(22.50,27.20) | 24.60(22.20,27.65) | 25.60(23.80,27.80) | 24.9(22.4,27.50) | Z=1.750 | 0.417 |
| **Grip strength (average)** |  | 23.90(19.25,31.55) | 20.00(16.14,24.57) | 16.60(12.25,22.36) | 21.6（17.33,29.02） | Z=47.977 | 0.001 |
| **Exercising** | No | 187（47.83） | 175（44.76） | 29（7.42） | 391 | χ2=15.122 | 0.001 |
|  |  |  |  |  |  |  |  |
|  | Yes | 102（66.23） | 46（29.87） | 6（3.90） | 154 |  |  |
| **Smoking** | Never | 201（51.67） | 164（42.16） | 24（6.17） | 389 | χ2=4.487 | 0.345 |
|  | Quitting | 34（65.38） | 14（26.92） | 4（7.69） | 52 |  |  |
|  | Yes | 54（51.92） | 43（41.35） | 7（6.73） | 104 |  |  |
| **Drinking** | no | 201（49.02） | 177（43.17） | 32（7.80） | 410 | χ2=12.733 | 0.002 |
|  | yes | 88（65.19） | 44（32.59） | 3（22.22） | 135 |  |  |
| **ADL score** |  | 14(14-14) | 14(14-14) | 14(14-20) | 14(14,4) | Z=30.716 | 0.001 |
| **SARC-F score** |  | 0(0-0) | 0(0-1) | 3(1-6) | 0(0,1) | Z=100.99 | 0.001 |
| **Education** | Illiterate | 40（24.39） | 105（64.02） | 19（11.59） | 164 | χ2=90.199 | 0.001 |
|  | Primary | 98（55.68） | 68（38.64） | 10（5.68） | 176 |  |  |
|  | else | 151（73.66） | 48（23.41） | 6（2.93） | 205 |  |  |
| **Marital status** | Spinsterhood | 4（40.00） | 4（40.00） | 2（20.00） | 10 | χ2=4.007 | 0.623 |
|  | Married | 253（53.83） | 188（40.00） | 29（6.17） | 470 |  |  |
|  | Widowed | 28（49.12） | 25（43.86） | 4（7.02） | 57 |  |  |
|  | Divorce | 4（50.00） | 4（50.00） | 0（0.00） | 8 |  |  |
| **Family income (yuan)** | ＜10000 | 33（43.42） | 37（48.68） | 5（6.58） | 76 | χ2=31.756 | 0.001 |
|  | 10000-19999 | 54（38.85） | 71（51.08） | 14（10.07） | 139 |  |  |
|  | 20000-34999 | 70（52.63） | 58（43.61） | 5（3.76） | 133 |  |  |
|  | ≥35000 | 132（66.67） | 55（27.78） | 11（5.56） | 198 |  |  |
| **Hypertension** | No | 126（56.25） | 88（39.29） | 10（4.46） | 224 | χ2=3.165 | 0.207 |
|  | Yes | 163（50.78） | 133（41.43） | 25（7.79） | 321 |  |  |
| **Coronary heart disease** | No | 241（56.57） | 164（38.50） | 21（4.93） | 426 | χ2=13.419 | 0.001 |
|  |  |  |  |  |  |  |  |
|  | Yes | 48（40.34） | 57（47.90） | 14（11.76） | 119 |  |  |
| **Operation** | No | 183（51.69） | 147（41.53） | 24（6.78） | 354 | χ2=0.776 | 0.677 |
|  | Yes | 106（55.50） | 74（38.74） | 11（5.76） | 191 |  |  |
| **Traumatism** | No | 278（53.36） | 213（40.88） | 30（5.76） | 521 | χ2=8.687 | 0.019 |
|  | Yes | 11（45.83） | 8（33.33） | 5（20.83） | 24 |  |  |

**Supplementary Table S4.** The characteristics among participants aged over 70 years old

| **Characteristic** |  | **Robust (n=142)** | **Pre-CF (n=151)** | **CF (n=47)** | **Total** | **test values** | **P-value** |
| --- | --- | --- | --- | --- | --- | --- | --- |
|  |  |  |  |  | **(n=340)** |  |  |
| **Sex** | Male | 80（48.19） | 75（45.18） | 11（6.63） | 166 | χ2=15.406 | ＜0.001 |
|  | Female | 62（35.63） | 76（43.68） | 36（20.69） | 174 |  |  |
| **BMI (kg/m^2^)** |  | 24.67±3.76 | 24.35±3.98 | 24.69±4.28 | 24.53±3.93 | F=0.292 | 0.747 |
| **Grip strength (average)** |  | 22.4(16.74.27.05) | 18.50(14.60,24.30) | 14.25(11.95,19.93) | 19.83(14.61,25.08) | Z=34.415 | ＜0.001 |
| **Exercising** | No | 97（38.80） | 112（44.80） | 41（16.40） | 250 | χ2=6.555 | 0.038 |
|  |  |  |  |  |  |  |  |
|  | Yes | 45（50.00） | 39（43.33） | 6（6.67） | 90 |  |  |
| **Smoking** | Never | 103（39.92） | 113（43.80） | 42（16.28） | 258 | χ2=5.903 | 0.203 |
|  | Quitting | 21（50.00） | 18（42.86） | 3（7.14） | 42 |  |  |
|  | Yes | 18（45.00） | 20（50.00） | 2（5.00） | 40 |  |  |
| **Drinking** | Never | 100（37.45） | 124（46.44） | 43（16.10） | 267 | χ2=11.459 | 0.061 |
|  | Sometime | 24（61.54） | 13（33.33） | 2（5.13） | 39 |  |  |
|  | Often | 5（50.00） | 4（40.00） | 1（10.00） | 10 |  |  |
|  | Every day | 13（54.17） | 10（41.67） | 1（4.17） | 24 |  |  |
| **ADL score** |  | 14(14,14) | 14(14,14) | 14(14,36) | 14(14,14) | Z=42.755 | ＜0.001 |
| **SARC-F score** |  | 0(0,1) | 0(0,2) | 4(2,6) | 0(0,2) | Z=78.728 | ＜0.001 |
| **Education** | Illiterate | 18（15.79） | 63（55.26） | 33（28.95） | 114 | χ2=64.349 | ＜0.001 |
|  | Primary | 68（50.37） | 57（42.22） | 10（7.41） | 135 |  |  |
|  | else | 56（61.54） | 31（34.07） | 4（4.40） | 91 |  |  |
| **Marital status** | Spinsterhood | 1（50.00） | 1（50.00） | 0 | 2 | χ2=4.028 | 0.68 |
|  | Married | 110（44.18） | 106（42.57） | 33（13.25） | 249 |  |  |
|  | Widowed | 27（33.33） | 41（50.62） | 13（16.05） | 81 |  |  |
|  | Divorce | 4（50.00） | 3（37.50） | 1（12.50） | 8 |  |  |
| **Family income (yuan)** | ＜10000 | 22（44.00） | 18（36.00） | 10（20.00） | 50 | χ2=7.699 | 0.261 |
|  | 10000-19999 | 24（34.78） | 33（47.83） | 12（17.39） | 69 |  |  |
|  | 20000-34999 | 22（36.67） | 28（46.67） | 10（16.67） | 60 |  |  |
|  | ≥35000 | 74（45.96） | 72（44.72） | 15（9.32） | 161 |  |  |
| **Hypertension** | No | 58（42.96） | 67（49.63） | 10（7.41） | 135 | χ2=8.117 | 0.017 |
|  | Yes | 84（40.98） | 84（40.98） | 37（18.05） | 205 |  |  |
| **Coronary heart disease** | No | 102（40.00） | 118（46.27） | 35（13.73） | 255 | χ2=1.565 | 0.457 |
|  | Yes | 40（47.06） | 33（38.82） | 12（14.12） | 85 |  |  |
| **Operation** | No | 86（40.57） | 98（46.23） | 28（13.21） | 212 | χ2=0.766 | 0.682 |
|  | Yes | 56（43.75） | 53（41.41） | 19（14.84） | 128 |  |  |
| **Traumatism** | No | 137（41.90） | 146（44.65） | 44（13.46） | 327 | χ2=1.227 | 0.581 |
|  | Yes | 5（38.46） | 5（38.46） | 3（23.08） | 13 |  |  |

**Supplementary Table S5.** The ordinal logistic regression among subgroup of participants aged 60-70 years old.

| **Exposure** | P | OR | 95%CI | |
| --- | --- | --- | --- | --- |
| **Robust** | 0.07 | 4.58 | 0.86 | 24.27 |
| **Pre-CF** | ＜0.001 | 159.29 | 27.82 | 912.17 |
| **female** | 0.06 | 1.65 | 0.99 | 2.77 |
| **Education (vs. Higher education)** |  |  |  |  |
| Illiterate | ＜0.001 | 5.37 | 3.22 | 8.97 |
| Primary | ＜0.001 | 2.14 | 1.33 | 3.43 |
| **Family income (yuan) (vs. ≥35000)** |  |  |  |  |
| ＜10000 | 0.06 | 1.78 | 0.96 | 3.27 |
| 10000-19999 | ＜0.001 | 2.10 | 1.28 | 3.43 |
| 20000-34999 | 0.39 | 1.25 | 0.75 | 2.07 |
| **Coronary heart disease (No vs. Yes)** | ＜0.001 | 0.50 | 0.32 | 0.78 |
| **Traumatism (No vs. Yes)** | 0.67 | 0.83 | 0.34 | 1.98 |
| **Exercising (No vs. Yes)** | ＜0.001 | 2.10 | 1.34 | 3.29 |
| **Drinking*** | 0.14 | 1.48 | 0.88 | 2.50 |
| **Grip strength (average)** | 0.01 | 0.96 | 0.93 | 0.99 |
| **ADL score** | 0.23 | 1.04 | 0.97 | 1.11 |
| **SARC-F score** | ＜0.001 | 1.55 | 1.34 | 1.79 |

* The drinking was classified as “Never or Sometime” compare to “often or everyday”

**Supplementary Table S6.** The ordinal logistic regression among subgroup of participants aged over 70 years old

|  | P | OR | 95%CI | |
| --- | --- | --- | --- | --- |
| **Robust** | 0.12 | 2.40 | 0.80 | 7.18 |
| **Pre-CF** | ＜0.001 | 57.06 | 16.96 | 191.93 |
| **Female** | 0.63 | 0.89 | 0.54 | 1.45 |
| **Education (vs. Higher education)** |  |  |  |  |
| Illiterate | ＜0.001 | 5.93 | 3.12 | 11.28 |
| Primary | 0.51 | 1.21 | 0.69 | 2.12 |
| **Hypertension (No vs. Yes)** | 0.81 | 0.95 | 0.60 | 1.49 |
| **Exercising (No vs. Yes)** | 0.96 | 1.01 | 0.61 | 1.69 |
| **Grip strength (average)** | 0.79 | 1.00 | 0.97 | 1.03 |
| **ADL score** | 0.35 | 1.02 | 0.98 | 1.07 |
| **SARC-F score** | ＜0.001 | 1.64 | 1.40 | 1.93 |

**Supplementary Table S7.** The multicolinearity assessment among participants aged 60-70 years old

| **multicolinearity assessment** | | |
| --- | --- | --- |
| **model** | Tolerance | VIF |
| **sex** | 0.47 | 2.12 |
| **Education** | 0.78 | 1.28 |
| **Marital status** | 0.92 | 1.08 |
| **Family income (yuan)** | 0.84 | 1.18 |
| **Hypertension** | 0.88 | 1.14 |
| **Coronary heart disease** | 0.89 | 1.12 |
| **Grip strength (average)** | 0.59 | 1.70 |
| **Exercising** | 0.94 | 1.06 |
| **Drinking** | 0.71 | 1.40 |
| **ADL score** | 0.64 | 1.56 |
| **SARC-F score** | 0.62 | 1.62 |
| **Operation** | 0.93 | 1.07 |
| **BMI (kg/m2)** | 0.89 | 1.12 |
| **Traumatism** | 0.95 | 1.05 |
| **Smoking** | 0.80 | 1.26 |

**Supplementary Table S8.** The multicolinearity assessment among participants aged over 70 years old

| **multicolinearity assessment** | | |
| --- | --- | --- |
| **model** | Tolerance | VIF |
| **sex** | 0.64 | 1.55 |
| **Education** | 0.80 | 1.25 |
| **Marital status** | 0.82 | 1.22 |
| **Family income (yuan)** | 0.77 | 1.30 |
| **Hypertension** | 0.88 | 1.14 |
| **Coronary heart disease** | 0.92 | 1.09 |
| **Grip strength (average)** | 0.69 | 1.45 |
| **Exercising** | 0.94 | 1.07 |
| **Drinking** | 0.73 | 1.37 |
| **ADL score** | 0.58 | 1.73 |
| **SARC-F score** | 0.53 | 1.88 |
| **Operation** | 0.92 | 1.08 |
| **BMI (kg/m2)** | 0.88 | 1.14 |
| **Traumatism** | 0.92 | 1.08 |
| **Smoking** | 0.76 | 1.32 |
